# Species Diversity and Antimicrobial Susceptibility of Staphylococci Colonising Healthy Dogs—A Single-Centre Cross-Sectional Study in Bulgaria

**DOI:** 10.3390/antibiotics15060536

**Published:** 2026-05-25

**Authors:** Velina Dinkova, Nikolina Rusenova

**Affiliations:** Department of Veterinary Microbiology, Infectious and Parasitic Diseases, Faculty of Veterinary Medicine, Trakia University, 6000 Stara Zagora, Bulgaria

**Keywords:** healthy dogs, commensal staphylococci, antimicrobial resistance, methicillin resistance, MIC, MALDI-TOF

## Abstract

**Background/Objectives**: Dogs are important carriers and transmitters of staphylococci from surface microbiota. Carriage screening allows for the identification of animals colonised with pathogens such as methicillin-resistant *S. pseudintermedius* (MRSP) and methicillin-resistant *S. aureus* (MRSA), which are spread between animals and from dogs to humans. This cross-sectional study determined the diversity of staphylococci from the surface microbiota of clinically healthy dogs in Bulgaria and their susceptibility to antimicrobial agents. **Methods**: The study was performed with 30 healthy dogs reared in the region of Stara Zagora, Bulgaria in 2024 and 2025. Swabs were obtained from eight body sites from each dog and incubated on blood and mannitol salt agar. Random isolates were identified by MALDI-TOF MS and tested for resistance to oxacillin/cefoxitin and to 14 classes of antimicrobial drugs (AMD). **Results**: Ninety out of 100 tested isolates were confirmed as *Staphylococcus* spp. from 15 different species. The total share of coagulase-positive (CoPS) staphylococci significantly exceeded that of coagulase-negative (CoNS) ones. Fifteen phenotypically methicillin-resistant staphylococci were identified—eight CoNS and seven CoPS—and confirmed by MIC test. The highest resistance was against penicillin (64.4%), ampicillin and minocycline (52.2%), whereas the highest sensitivity was to rifampin, amikacin, cefquinome and amoxicillin + clavulanic acid. **Conclusions**: Data about the carriage of MRSP, MRSA and multidrug-resistant coagulase-negative staphylococci in healthy dogs are important in view of the increased risk of colonisation/infection for people in contact with these dogs in households and veterinary facilities (clinics, hospitals). This supports the “One Health” approach integrating animal, human and environmental health.

## 1. Introduction

Knowledge about staphylococci colonization in healthy dogs is important for a number of reasons. Although part of the normal skin and mucosal microbiota, they can become pathogenic when the integrity of the skin barrier is impaired after inoculation and in immunocompromised patients. Dogs have the highest skin pH of all domestic animals—about 7.4 (8.62–6.84). This favours the development of staphylococci and *Pseudomonas aeruginosa* (with optimum pH for development between 7 and 7.5)—common causes of infections from the resident microbiota in this animal species [1,2]. Staphylococci are resistant to environmental changes, and possess diverse attack mechanisms and strategies for avoiding and manipulating a host’s defense mechanisms [3].

The role of different *Staphylococcus* species as commensal, opportunistic or pathogenic microorganisms in dogs is still under evaluation. Although coagulase-positive *S. pseudintermedius* is by far the most commonly detected species in both healthy and diseased dogs [4,5], *S. aureus*, *S. schleiferi*, *S. epidermidis* and *S. haemolyticus* also pose a challenge to small animal clinical practice [6,7].

Humans and dogs share not only living environments but also antibiotics, a major factor in the emergence of antimicrobial resistance (AMR) worldwide [8]. AMR in animals is associated with three groups of bacteria: specific animal pathogens, zoonotic pathogens and commensal bacteria. The commensal group is of particular importance due to its significantly higher biomass compared to the other two groups [9]. Dogs maintain staphylococci from surface microbiota as reservoirs, carriers and transmitters. From this perspective, carriage screening allows for the identification of animals colonised with dangerous pathogens such as methicillin-resistant *S. pseudintermedius* (MRSP) and methicillin-resistant *S. aureus* (MRSA), which are spread between animals and from dogs to humans [10]. Staphylococci rapidly become resistant to almost all major classes of antimicrobial agents, so even the coagulase-negative staphylococci previously considered non-pathogenic deserve attention due to their potential to acquire and spread resistance genes [11]. Data on staphylococcal carriage in healthy dogs are also useful for veterinary facilities (clinics, kennels) supporting the “One Health” approach integrating animal, human and environmental health.

Published data on carriage of staphylococci by healthy dogs are extremely diverse due to differences in culturing and identification methods, dog breeds, anatomical site of the sample, living conditions and geographical location. In Bulgaria, no specific screening of *Staphylococcus* spp. carriage in dogs has been conducted; therefore, the present cross-sectional study aimed to determine the diversity of staphylococcal species from the surface microbiota of clinically healthy dogs raised under different conditions in the Stara Zagora region, Bulgaria and their susceptibility to antimicrobial agents.

## 2. Results

Out of all the 240 examined swab samples, 17 were negative. A total of 197 presumptive *Staphylococcus* spp. were identified by classical bacteriological methods (cell and colony morphology, Gram staining, catalase and oxidase tests). From 100 selected isolates, MALDI-TOF confirmed 90 as staphylococci belonging to 15 different species (Table 1). Eight from the rest of the 10 isolates were not reliably identified, and two were determined to be *Mammaliicoccus sciuri*.

The randomly selected set included 43 skin isolates and 47 from mucosal/mucocutaneous sites (*p* = 0.752). There were no statistically significant differences in the distribution of isolates in terms of dog sex (*p* = 0.09) or living environment (*p* = 0.07). Also, the distribution of isolates according to the body site did not demonstrate significant predominance of strains from any of the eight anatomical sites (*p* = 0.499; Table S1). The most frequently encountered species were *S*. *pseudintermedius* (n = 49; 54.4%), *S*. *haemolyticus* (n = 8; 8.9%), *S*. *epidermidis* (n = 7; 7.8%), *S*. *aureus* (n = 5; 5.6%) and *S*. *simulans* (n = 5; 5.6%) (Table 1).

The proportion of coagulase-positive (CoPS) staphylococci significantly exceeded that of coagulase-negative (CoNS) ones—62.2% vs. 37.8%; *p* = 0.027. CoPS and CoNS were almost evenly distributed on the skin (52.4% and 47.6%, respectively) unlike on mucous membranes (70.8% vs. 29.2%). A different distribution was also established in terms of the living environment of dogs. The CoPS and CoNS colonising animals raised in the university biobase and outdoor in yards were 55.6% and 44.4% of all isolates, respectively, whereas the CoPS proportion in indoor pets was substantially greater—72.2% (*p* = 0.003) compared to 27.8% for CoNS.

**Figure 1:**
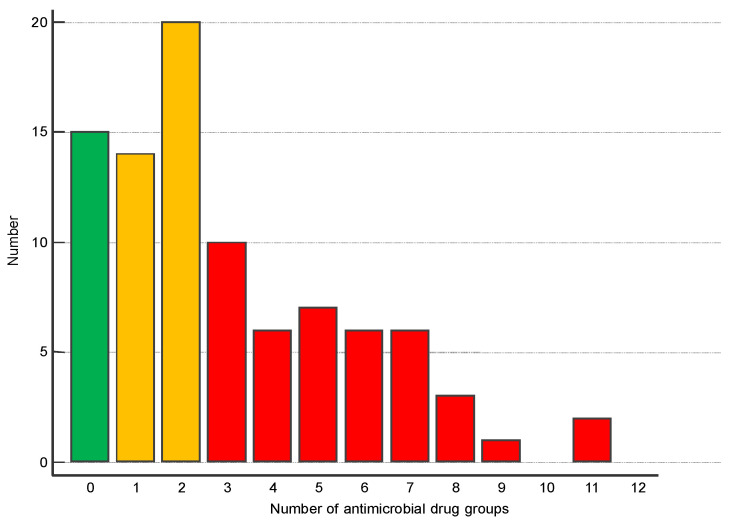
Distribution of staphylococci colonising healthy dogs according to the number of anti- microbial drug (AMD) groups to which they were resistant.

Of all the 90 isolates, 15 were susceptible to all tested antimicrobial drugs (AMD), and 34 (37.8%) were resistant to one or two AMD groups. The remaining 45.6% were multidrug-resistant (MDR) (Figure 1).

The behaviour of the isolates to each of the tested AMDs is presented in Table 2. The highest resistance was found against penicillin (64.4%), ampicillin and minocycline (52.2%). Of all the isolates, only one was resistant to rifampin, two to amikacin and three to cefquinome and amoxicillin + clavulanic acid.

**Figure 2:**
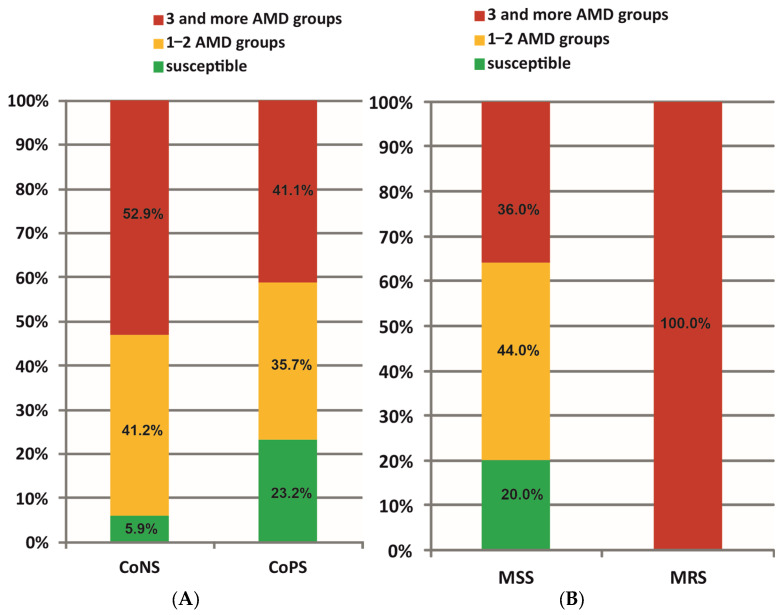
Distribution of CoNS/CoPS (**A**) and MSS/MRS staphylococci (**B**) colonising healthy dogs according to their sensitivity to antimicrobial drugs (AMD).

Figure 2 illustrates the distribution of CoPS and CoNS, as well as MRS and MSS staphylococci according to the number of AMD groups. Multiresistant CoPS were 41.1% of all isolates from this group, whereas the proportion of multiresistant CoNS was insignificantly greater—52.9% (*p* = 0.384). CoPS and CoNS showed similar susceptibility, being resistant against one to 11 AMD groups (median three). A significant difference (*p* < 0.001) was found in the number of AMD groups to which MRS and MSS showed resistance: from three to 11 (median six) and from one to nine (median two), respectively.

**Figure 3:**
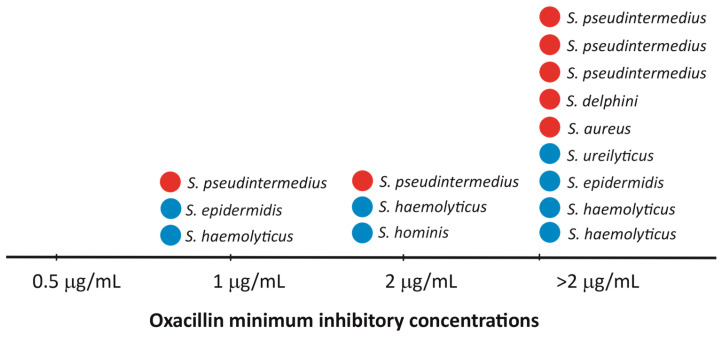
Distribution of oxacillin MIC values of 15 phenotypically methicillin-resistant *Staphylococcus* sp. isolates. Red points correspond to coagulase-positive and blue points: to coagulase-negative species.

Phenotypically methicillin-resistant staphylococci (MRS) were 15 (16.7%)—eight CoNS and seven CoPS. Most MR CoPS were from the *S. pseudintermedius* species (five/seven), whereas half of MDR CoNS were *S. haemolyticus* (four/eight). The oxacillin MIC values confirmed the results from the disc diffusion testing (Figure 3). The distribution of MICs to all tested AMD is shown in Table 3.

## 3. Discussion

### 3.1. Carriage Rates

Coagulase-negative staphylococci (CoNS) are well adapted to long-term colonisation of healthy skin and are consistently present in healthy dogs. Coagulase-positive staphylococci are also part of normal flora but at lower abundance and at specific sites (nostrils, perineum) [14]. The intact skin barrier and healthy immune function favour CoNS, which coexist without damaging tissues.

A number of factors influence the prevalence of staphylococcal species in normal canine microbiota. First, the distribution varies by anatomical location. In the present study, CoPS and CoNS were almost evenly distributed on the skin (53.5% and 46.5%, respectively) unlike on mucosal surfaces (70.2% vs. 29.8%). This may be attributed to the conditions in the different body sites: CoNS are more abundant on dry skin, being more tolerant to desiccation; moist areas with more nutrients (perineum) harbour both CoPS and CoNS; whereas CoPS predominate on mucosal surfaces due to their better adherence and colonisation ability [15]. In the present study, this is particularly demonstrated by the fact that 80% of *S. aureus* strains were isolated from mucosae.

In relation to the living environment of dogs, CoPS prevailed in home-owned dogs (72.2%). This may be explained by the fact that indoor dogs are also exposed to microorganisms specific to humans and the home environment. According to several molecular typing reports, dogs and cats are colonised/infected with MRSA by humans [16]. In our study, MALDI-TOF identified one of the five *S. aureus* isolates as belonging to the CC8 lineage. An earlier study conducted in France from 2010 to 2015 also demonstrated that cats and dogs were mainly infected by human-related MRSA clones corresponding to the human MRSA epidemiology such as CC5 and CC8 [17]. The MRSA diversity within the companion animal population in Austria showed that although ST398 isolates remained predominant, other human-associated MRSA clones were observed with CC8 prevalence in two out of ten dogs (20%) [18]. Also, different grooming habits, such as regular washing and bathing, may be responsible for the removal of CoNS and CoPS expansion in home-owned dogs. It is reported that enhanced *S. aureus* skin colonisation correlates with a loss of microbiome diversity [19]. Some commensal CoNS directly counter both the carriage and invasion of the common dermatopathogen *S. aureus* [20] and can actively suppress the growth of CoPS [21].

The skin microbiota in the different cutaneous and mucocutaneous regions in healthy dogs shows significant site variability. Higher microbial diversity was observed in the haired skin (axilla, groin, pinna, dorsal nose) compared to mucosal surfaces (mouth, nose, conjunctiva) and mucocutaneous junctions. The nostrils and conjunctiva showed the lowest, while the axilla and dorsal nose showed highest microbial diversity [15]. Our results are in line with these findings, showing only three staphylococcal species in samples from the oral and nasal cavities and prepuce/vulva and only four species in conjunctival samples, whereas the skin of the groin and axilla harboured eight and seven species, respectively (Table S1). The mouth, nostrils and the perianal mucocutaneous site serve as a reservoir for *S. pseudintermedius* colonisation of the skin and hair in dogs through normal grooming activity. The ear is another site where *S. pseudintermedius* is frequently isolated [14].

The existing data on the prevalence and diversity of commensal canine *Staphylococcus* spp. are rather various. In some reports, coagulase-positive species predominate similarly to our study. CoPS were isolated from 74% of healthy dogs by Griffeth et al. [22]: *S*. *aureus* (16%), *S. pseudintermedius* (92%) and *S. coagulans* (5%). Miszczak et al. [5] investigated the presence of *Staphylococcus* spp. in samples from the external ear canal, conjunctivae, nostrils, mouth, groin and anus in 113 dogs. The commonest species was *S. pseudintermedius* (53.28%)—a rate comparable to that in our study, followed by *S. epidermidis* (16.22%), which ranked third in prevalence in our sample.

In Valencia, Spain, the ratio between CoPS and CoNS in healthy animals was 79.8% to 20.2%. The first group comprised *S. aureus* (7.1%), *S. pseudintermedius* (64.2%) and *S. coagulans* (8.3%). The second group included *S. cohnii*, *S. epidermidis*, *S. haemolyticus*, *S. hominis*, *S. sciuri*, *S. simulans*, *S. warneri* and *S. xylosus* with prevalence rates between 1.2% and 4–8% [23]. In nasal swab samples of 40 healthy dogs, Lan Anh et al. [24] recovered *Staphylococcus* spp. from 47.5% of animals. *S. aureus*, *S. pseudintermedius* and *S. epidermidis* constituted 80.46% of strains. Comparable to our results, the share of *S. pseudintermedius* was the greatest (48.28%).

Wilkinson et al. [25] isolated *S*. *pseudintermedius* from buccal and perianal swabs of 92/126 (73.0%) clinically healthy dogs referred for castration or routine vaccination in veterinary clinics. Out of 119 staphylococci isolated from healthy stray dogs in Naples, Italy, *S. pseudintermedius* was the most frequent species (50%), followed by *S. simulans* (17%) and *S. aureus* (14%) [26]. In our survey, these three species were represented with 54.4%, 5.6% and 5.6%, respectively.

In other publications, the normal staphylococcal population in healthy dogs included more coagulase-negative species. Schmidt et al. [27] identified 436 staphylococci in nasal and perineal samples from Labrador retrievers; of them, 102 were CoPS from two species and 334 were CoNS from 18 species. *S. pseudintermedius* was the second most common species and the most prevalent CoPS. The predominant recovered CoNS was *S. epidermidis* in 52% of dogs, contrary to data from Garbacz et al. [28] who did not report *S. epidermidis* in dogs. Gandolfi-Decristophoris et al. [29] investigated the distribution of commensal staphylococcal species in nostrils and ears of 256 healthy dogs where CoNS species accounted for 60% (172/284) of all isolates. The diversity of coagulase-negative staphylococci (CoNS) was high—22 species, with *S. epidermidis* and *S. haemolyticus* being the most encountered. *S. pseudintermedius* was the most isolated CoPS species. In our study, *S. epidermidis* ranked third by prevalence with 7.8%, after *S. pseudintermedius* and *S. haemolyticus*.

Newstead [30] examined swabs from different body sites of 121 healthy dogs in Sweden and Scotland and reported a total of 18 *Staphylococcus* species, 36.4% of them belonging to three CoPS species (*S*. *pseudintermedius*, *S*. *aureus*, *S*. *coagulans*). The present study also identified three CoPS species. In nasal, buccal and perineal samples from dogs in Trinidad, Suepaul et al. [31] detected 24 staphylococcal species, half of which were CoNS (50.2%), 41.7% were CoPS and 8.1% were coagulase-variable. Frequently isolated CoPS were from the SIG group (38.7%), followed by *S. aureus* (2.1%) and *S. lutrae* (0.9%). Predominant CoNS species were *S. sciuri* and *S. simulans*—26.4% and 13.6% of all isolates, respectively.

### 3.2. Sensitivity to Antimicrobial Drugs

Several types of antimicrobial resistance are observed in bacteria. Intrinsic AMR is the result of spontaneous mutations or acquisition of new genetic material, making microorganisms insensitive to a given antibiotic without the need for additional resistance factors. In adaptive resistance, which is transient, gene expression is altered due to the presence of antimicrobial drugs. However, bacteria with intrinsic or adaptive resistance are not considered a threat in the context of the global spread of AMR [32]. A serious problem is the so-called acquired resistance—an evolutionary phenomenon that allows microorganisms to develop mechanisms for protection against other bacteria or antimicrobial drugs.

A given microorganism can exhibit multiple resistance mechanisms, especially in environments with high bacterial load, e.g., soil and healthcare facilities, but they can be also acquired through pets [33]. The antibiotic resistance pattern observed in cats and dogs is related to the amount and frequency of use of certain antibiotics. The findings of Brookshire et al. [34] strongly suggest that in the absence of risk factors for resistance (recent antibiotic use or previous resistant infections), dogs are much more likely to be susceptible to a broad spectrum of AMD than animals that have already been treated.

Several groups of resistant bacteria are transmitted from pets to humans, posing a serious threat to public health. Among staphylococci, these are methicillin-resistant coagulase-positive isolates [10]. Therefore, many studies in different countries have investigated their prevalence and antimicrobial resistance.

In the present study, 16.7% of all 90 tested isolates were phenotypically methicillin-resistant—7/56 CoPS (12.5%) and 8/34 (23.5%) CoNS. Reported carriage rates of methicillin-resistant *S. aureus* (MRSA) and *S. pseudintermedius* (MRSP) vary considerably. Low rates were obtained for isolates from five sites in 50 healthy dogs where MRSA was not detected, and the occurrence of MRSP was 3% [22]. Similarly, Bean & Wigmore [35] obtained ear, buccal, nasal and perineal swabs from 117 healthy dogs and detected two MRSP and no MRSA. The most common resistance was to penicillin (95.2% in *S. aureus* and 72.4% in *S. pseudintermedius*) and doxycycline/tetracycline (19.7% in *S. pseudintermedius*). The rate of resistance to the other tested antimicrobials was low. In a random sample of nasal swabs from healthy pet dogs in Hong Kong, Epstein et al. [36] did not find MRSA, but reported a higher prevalence of MRSP (17%).

In Germany, methicillin resistance, evidenced by the *mecA* gene, was found in six *S. aureus* colonising 5/192 (2.6%) dogs [37]. In contrast, having studied the prevalence of MRSA in healthy dogs from the Kurdistan region of Iraq, Abdulrahman [38] reported a carriage rate of 27% (4/15), higher than the phen-MRSA rate of 20% in our study. No MRSP was identified in clinically healthy dogs by Rubin & Chirino-Trejo [39] in Canada, Wilkinson et al. [25] in New Zealand and Popa et al. [40] in Timisoara, Romania. The absence of MRSP in these three studies may indicate a relatively low prevalence of methicillin resistance in *S. pseudintermedius* among healthy dog populations, at least in the studied regions. Viñes et al. [41] identified 27 MSSP (40%) and 40 MRSP (60%); 29 of the latter were MDR. Of the 49 *S. pseudintermedius* identified in our screening study, the proportion of phen-MRSP was 10.2%—similar to that in the research of Naziri & Majlesi [42], where the MRSP carriage rate in healthy dogs was 12%. Regarding multidrug-resistant *S. pseudintermedius*, their proportion in our survey exceeds that of Ferrer et al. [43] who reported that only two of 22 *S. pseudintermedius* isolated from the skin of healthy dogs (9.1%) were MDR. Among our MSSP isolates, 31.8% were MDR.

Štempelová et al. [4] characterised staphylococci from the inner ear, chin, nasal skin, back, axilla, abdomen, interdigital and perianal areas in healthy dogs. The most common resistance observed in a total of 91 staphylococcal isolates was to chloramphenicol (73%), penicillin (67%), erythromycin (42%) and tetracycline (26%). All strains were sensitive to gentamicin and vancomycin. Multidrug resistance was found in 50% of the isolates.

Susceptibility to all AMDs tested was the most common phenotype identified in 46.4% of *S. pseudintermedius* isolated from 153 dogs [43]. In this study, no resistance to ceftiofur, moxifloxacin, ciprofloxacin, enrofloxacin, chloramphenicol, rifampin, nitrofurantoin or vancomycin was found. In our survey, seven vancomycin-resistant CoPS strains were identified. In general, drug susceptibility to some last resort antibiotics, e.g., vancomycin and nitrofurantoin, authorised for human use only is very high. Marco-Fuertes et al. [23] established a resistance rate to vancomycin ranging from 0% in *S. pseudintermedius* to 50% in *S. aureus* in healthy dogs. Thus, the identification of vancomycin resistant canine staphylococci is an important issue within the One Health context as they may threaten public health through the transfer of resistance genes to bacteria colonising humans.

Penicillin and tetracycline resistance were the most common, in 39.9% and 23.5% of isolates, respectively, although half of the penicillin-resistant (30 of 61) and tetracycline-resistant (18 of 36) isolates were resistant to only one agent. No isolate was simultaneously resistant to β-lactams, tetracyclines and macrolides, confirming the utility of these therapeutic options [40].

In *S. pseudintermedius* from healthy dogs, MIC tests determined resistance to penicillin in 82.1% of isolates, to tetracycline in 71.6%, to ampicillin in 59.7%, to clindamycin in 43.3%, to co-trimoxazole in 32.8% and to gentamicin in 10.5%, and 100% susceptibility to rifampin [41]. In our study, the resistance of *S. pseudintermedius* to most antibiotics was lower: 51% to penicillin; 30.6% to tetracycline, 45% to ampicillin and 18% to clindamycin. Susceptibility to sulfamethoxazole/trimethoprim was similar, and all isolates except one were susceptible to rifampin. Elnageh et al. [44] also noted 100% susceptibility to rifampin.

Of a total of 78 presumptive *Staphylococcus* spp. from 48 healthy dogs and 30 cats in southern Brazil during 2018–2022 [7], 25% of isolates were susceptible to all tested agents, and the rest were susceptible to one to 12 antimicrobials (mean 3; median 3). In addition, 61.8% were resistant to penicillin and 20.6% to azithromycin and doxycycline. Resistance to the remaining AMDs tested was less than 20%. Of the isolates classified as MDR, 35.3% showed resistance to three to eight classes; 13 (19.1%) were methicillin-resistant. The most frequently observed MDR patterns were beta-lactams + macrolides + tetracyclines, beta-lactams + tetracyclines + sulfonamides and beta-lactams + lincosamides + macrolides + tetracyclines + sulfonamides. In the same study, eight CoPS isolates (30.8%) were susceptible to all antimicrobials and 18 (69.2%)—to one to nine AMD (mean 2.6, median 1.5). *S. pseudintermedius* was isolated most frequently (n = 20; 77%), of which 14 were resistant to one to nine AMD (mean 3.1; median 2.5). A similar picture was obtained for *S. pseudintermedius* in our study—resistance to one to nine AMD groups (median 3).

Fàbregas et al. [45] studied *S. pseudintermedius* populations colonising the skin of healthy dogs and identified both MRSP and MSSP genotypes. Based on the detection of the *mecA* gene, 39% of *S. pseudintermedius* were resistant to methicillin. MRSP isolates showed a higher number of antibiotic resistance genes (three to 10) compared to MSSP isolates (one to two). In our study, the proportion of phen-MRSP among all *S. pseudintermedius* was much lower (10.2%), but their behaviour towards AMD was similar—resistance to one to nine AMD groups (median two) for MSSP and to four to seven AMD groups (median seven) for phen-MRSP.

Nocera et al. [26] studied resistant *Staphylococcus* spp. in the ear and nose of stray dogs housed at the Veterinary Hospital of Naples. Antimicrobial resistance profiles of 119 *Staphylococcus* spp. isolates revealed significant circulation of methicillin-resistant strains. The highest levels of resistance were observed for penicillins (85.7%), cephalosporins (75.6%) and tetracyclines (61.3%), while the lowest levels were recorded for aminoglycosides (21.8%), fluoroquinolones (30.3%) and sulfonamides (31.9%). No resistance to vancomycin was recorded.

All *S. pseudintermedius* from the ears of 360 healthy dogs in Romania tested against 14 antimicrobials from 11 classes between 2022 and 2025 were susceptible to glycopeptides, oxazolidinones, fusidanes and glycylcyclines [40]. They showed the highest resistance to tetracycline—25%; 19.7% were resistant to β-lactams and 22.4% to lincosamides. González-Martín et al. [46] determined the frequency of nasal carriage of CoPS among healthy dogs in Spain. *S. aureus* carriage was found in 5.9%; *S. pseudintermedius* in 32.4% of the dogs; both species harbored resistance genes or mutations associated with nine classes of antimicrobials. The antimicrobial resistance patterns of *S. pseudintermedius* isolates were: penicillin (57.9%), tetracycline (26.3%), trimethoprim–sulfamethoxazole (63.2%), and chloramphenicol (5.3%). These levels could reflect their widespread and long-term use and associated constant selective pressure over time.

The reviewed results on the presence of MDR isolates indicate that clinically healthy dogs can act as important reservoirs of resistant bacteria with potential zoonotic consequences. These data highlight the urgent need for reliable antimicrobial resistance surveillance strategies and prudent antibiotic use in veterinary medicine to limit the spread of resistant strains and protect both animal and public health.

In general, published data on coagulase-negative staphylococci in healthy dogs indicate that a significant proportion of them were methicillin-resistant (MR). In healthy Labrador retrievers, Schmidt et al. [27] found no MR-CoPS, but MR-CoNS were detected in 42% of dogs, even in animals without prior antimicrobial treatment or a visit to a veterinary clinic. From a total of 38 staphylococci isolated from 14 healthy dogs, 24 were CoNS and 14 CoPS [44], and a methicillin-resistant phenotype was expressed by 11 CoNS and two *S. aureus*. Another study assessed the prevalence of coagulase-negative staphylococci in 50 clinically healthy dogs and found that 8.5% expressed the *mecA* gene [47]. Antimicrobial susceptibility testing results showed that 23.7% were resistant to penicillin, 22% to ampicillin and 16.9% to erythromycin. In our study, CoNS isolates showed substantially higher levels of resistance to these three antibiotics: 82.4%, 61.8% and 47.1%, respectively. These data are of concern because of the potential for opportunistic infections, and transfer of mobile genetic elements of antimicrobial resistance from these bacteria to coagulase-positive staphylococci.

In CoNS (n = 42) isolated from healthy pets in Brazil [7], susceptibility testing showed that apart from the nine susceptible strains, the remaining isolates were resistant to one to 12 AMD (mean 2.8; median 2.5); 59.5% of the isolates were resistant to penicillin, while resistance to the other antimicrobials tested was less than 27%. The comparative analysis among the eight identified CoNS species indicated that *S. equorum* was resistant to three to 12 of the 16 antimicrobials tested (mean 5.4; median 4.5), followed by *S. sciuri* with resistance to one to six antimicrobials (mean 2.6; median two). In our study, the most commonly isolated CoNS were *S. haemolyticus*, resistant to two to 11 AMD groups (median five) and *S. epidermidis*—resistant to one to six AMD groups (median 3.5).

The present study has limitations that should be mentioned: the small number of sampled animals, the cross-sectional single-centre study design, and the lack of molecular confirmation of genes conferring methicillin resistance. The study provides a snapshot of the current situation in the studied region, so the reported data are of local significance and should not be considered representative of larger populations. Future studies using molecular techniques, including temporal ones, will be necessary to obtain a more comprehensive picture on commensal microbiota of dogs for more correct assessment of the risk of infection for people in contact with them.

In conclusion, on the skin and mucosae of dogs, staphylococci share a common ecological niche with a wide range of Gram-positive and Gram-negative species, and this polymicrobial environment promotes the easy acquisition of resistance genes. The results of the present study confirm that a significant proportion of commensal staphylococci inhabiting the normal skin and mucous membranes of healthy dogs are multidrug-resistant. Monitoring for carriage of MRSA, MRSP, and multidrug-resistant coagulase-negative staphylococci is important in view of the increased risk of colonisation/infection for people in contact with these dogs in households, schools, and hospitals.

## 4. Materials and Methods

### 4.1. Animals and Samples

The study cohort included six healthy dogs housed at the Biobase of the Faculty of Veterinary Medicine (FVM), Stara Zagora, and 24 privately owned dogs referred to the University Veterinary Hospital (UVH) of the FVM for diagnostic imaging, castrations or orthopaedic surgery during July–August 2024 and July–August 2025. The animals were not treated with antibiotics for at least one month. The study was approved by Protocol No 8-2023 of 3 July 2023 of the Ethical Committee of the FVM and by written permit of the UVH Manager, and informed consent was obtained from all owners.

Eight swabs were obtained from each of the dogs: from the nasal and oral cavity, conjunctiva, external ear canal, vulva/prepuce, axilla, groin and perianal areas. The anatomical sites were chosen to represent different microbial habitats: mostly dry haired skin (axilla, groin), mucous membranes (nasal and oral cavity, conjunctiva, vulva/prepuce) and mucocutaneous areas (perianal area at the skin-mucosal interface). Each site was swabbed for 5–10 s with sterile cotton applicators, transferred to transport medium (Deltalab, Barcelona, Spain) and processed in the bacteriological laboratory within 24 h.

### 4.2. Microbiological Examination and Identification of Isolates

Collected swabs were plated on blood agar with 5% defibrinated sheep blood, and mannitol salt agar is a selective growth medium to isolate *Staphylococcus* spp. Samples were aerobically incubated for 18–24 h at 37 °C. After the initial incubation, the plates were checked for the presence of bacterial growth. If no visible growth was detected, incubation continued for another 24 h at 37 °C. The primary identification was based on clinical veterinary microbiology guidelines [48]. The diagnostic algorithm for Gram-positive cocci included Gram staining, catalase and oxidase activity tests, and additional tests if necessary.

One hundred presumptive staphylococcal isolates were for identification to the species level by matrix-assisted laser desorption/ionization time-of-flight mass spectrometry (MALDI-TOF MS) automated system. The inclusion strategy included random selection of half of presumptive staphylococci from every animal (i.e., nearly every second isolate) by a random number generator (https://www.calculatorsoup.com/calculators/statistics/random-number-generator.php accessed on 8 May 2026) so that strains from all dogs were represented. The stratification of the final set of identified 90 isolates was statistically tested to ensure that there were no significant differences in terms of the strains’ origins. Single colonies from 24-h pure culture on blood agar (37 °C; aerobic conditions) were inoculated on reusable plates and placed in the MALDI-TOF MS instrument (Bruker Daltonics, Bremen, Germany) for analysis. Spectra were analysed automatically and the matches of the generated peak lists to the reference library data using the BioTyper software v. 4.1.100 were evaluated using a logarithmic score. A score of 2.0 validated the identification to the species level, scores between 1.7 and 2 corresponded to a reliable identification of the genus and those with scores below 1.7 were not used.

### 4.3. Antimicrobial Sensitivity Testing

Screening for methicillin sensitivity was performed on Mueller–Hinton agar plates incubated at 35 °C for 24 h according to the criteria for sensitivity of different staphylococcal species to cefoxitin (30 µg, MASTDISCS, Mast Group Ltd., Merseyside, UK) for *S. aureus* and coagulase-negative species, or to oxacillin (1 µg, MASTDISCS, Mast Group Ltd., UK) for *S. pseudintermedius* and *S. schleiferi* [13].

All isolates were tested against 25 antimicrobial drugs (AMD) from 14 classes by the Kirby–Bauer disc diffusion method as per CLSI M100 and CLSI VET01S [12,13]. Penicillins were represented by penicillin (10 µg) and ampicillin (10 µg); potentiated penicillins—with the combination amoxicillin/clavulanic acid (20/10 µg, MASTDISCS, Mast Group Ltd., UK) and carbapenems—were represented by imipenem (10 µg, MASTDISCS, Mast Group Ltd., UK). Cephalosporins included first-generation cephalexin (30 µg) and cefazolin (30 µg), third-generation cefotaxime (30 µg) and fourth-generation cefquinome (30 µg, Biolab, Budapest, Hungary). Tested fluoroquinolones included enrofloxacin (5 µg), marbofloxacin (5 µg, Biolab, Hungary) and pradofloxacin (5 µg, Biolab, Hungary); aminoglycosides: gentamicin (10 µg) and amikacin (30 µg); macrolides: erythromycin (15 µg) and azithromycin (15 µg); lincosamides: clindamycin (2 µg); tetracyclines: tetracycline (30 µg), doxycycline (30 µg) and minocycline (30 µg); glycopeptides: vancomycin (30 µg); amphenicols: chloramphenicol (30 µg); ansamycins: rifampin (5 µg); nitrofuran derivatives: nitrofurantoin (100 µg) and potentiated sulfonamides: sulfamethoxazole/trimethoprim (23.75/1.25 µg, MASTDISCS, Mast Group Ltd., UK). All other discs were manufactured by HiMedia (Thane, India). The reference strain *Staphylococcus aureus* ATCC 25923 was used as internal quality control of antimicrobial sensitivity zones.

The minimum inhibitory concentrations (MICs) of all methicillin-resistant isolates were determined by broth microdilution (Sensititre Vet Companion Animal Gram Positive Plate with Pradofloxacin COMPGP1F, Thermo Fisher Scientific, Waltham, MA, USA) for 23 AMD and two AMD combinations. The bacterial isolates from a single colony by overnight culture, adjusted to a final concentration of 0.5 MacFarland standard in Mueller–Hinton broth, was added to Sensititre plates and incubated at 35 °C for 24 h. The endpoints were read using Sensititre Manual Viewbox (Thermo Fisher Scientific, USA). The range of the AMDs in the plates were as follows: 0.25–2 µg/mL for oxacillin, 0.06–8 µg/mL for penicillin, 0.25–8 µg/mL for ampicillin, 0.25/0.12–8/4 µg/mL for amoxicillin/clavulanic acid, 1–4 µg/mL for imipenem, 0.25–4 µg/mL for enrofloxacin, 1–4 µg/mL for marbofloxacin, 0.25–2 µg/mL for pradofloxacin, 2–4 µg/mL for cefazolin, 0.06–8 µg/mL for cefovecin, 2–4 µg/mL for cephalotin, 2–8 µg/mL for cefpodoxime, 4–16 µg/mL for gentamicin, 16–32 µg/mL for amikacin, 0.25–4 µg/mL for erythromycin, 0.5–4 µg/mL for clindamycin, 0.25–1 µg/mL for tetracycline, 0.12–0.5 µg/mL for doxycycline, 0.5–2 µg/mL for minocycline, 1–16 µg/mL for vancomycin, 8–32 µg/mL for chloramphenicol, 1–2 µg/mL for rifampin, 16–64 µg/mL for nitrofurantoin and 2/38–4/76 µg/mL for sulfamethoxazole/trimethoprim. Results were interpreted following species-specific breakpoints outlined in CLSI VET01S [13]; when dog-specific breakpoints were not available, human breakpoints listed in CLSI M100 [12] were used. For MIC_90_ calculation, the lowest MIC value was used for isolates without growth in any of the offered concentrations, whereas the next higher serial MIC value was assigned to strains demonstrating growth in all tested concentrations. The reference strain *Staphylococcus aureus* ATCC 29213 served for internal quality control for MIC ranges.

Isolates were classified as multiresistant (to at least one antimirobial agent from three or more AMD classes) according to Magiorakos et al. [49].

### 4.4. Statistical Analysis

The information was organised into spreadsheets for descriptive analysis. Data were dichotomised by site of sample collection (haired skin, mucous membranes and mucocutaneous areas), sex (male, female), living environment of the dog (outdoor, indoor), and antimicrobial susceptibility of the isolate (susceptible or resistant; the strains with intermediate susceptibility were considered resistant). The non-parametric Mann–Whitney test was used to test the differences in the median number of AMD groups between between CoPS and CoNS and between MRS and MSS. The two independent proportions and chi-square tests were applied for comparison of proportions of independent groups of samples using MedCalc v.15.8 (MedCalc Software bvba, Ostend, Belgium). *p*-values less than 0.05 were considered statistically significant.

## Figures and Tables

**Table 1:** Species diversity of staphylococci (n = 90) isolated from healthy dogs.

Species	Number	Percentage
*Staphylococcus pseudintermedius*	49	54.4%
*Staphylococcus aureus*	5	5.6%
*Staphylococcus delphini*	2	2.2%
*Staphylococcus haemolyticus*	8	8.9%
*Staphylococcus epidermidis*	7	7.8%
*Staphylococcus simulans*	5	5.6%
*Staphylococcus warneri*	3	3.3%
*Staphylococcus hominis*	2	2.2%
*Staphylococcus lugdunensis*	2	2.2%
*Staphylococcus ureilyticus*	2	2.2%
*Staphylococcus caprae*	1	1.1%
*Staphylococcus saprophyticus*	1	1.1%
*Staphylococcus schleiferi*	1	1.1%
*Staphylococcus simiae*	1	1.1%
*Staphylococcus succinus*	1	1.1%
Total	90	100.0%

**Table 2:** Percentages of resistance of staphylococci (n = 90) isolated from healthy dogs to tested antimicrobial drugs (AMD).

AMD Groups	Tested AMD	Number of Resistant Isolates (%; 95% CI)
Penicillins	Penicillin (10 µg)	58 (64.4%; 48.9–83.3%)
	Ampicillin (10 µg)	47 (52.2%; 38.4–69.4%)
Potentiated penicillins	Amoxicillin/clavulanic acid (20/10 µg)	3 (3.3%; 0.7–9.7%)
Carbapenems	Imipenem (10 µg)	-
Cephalosporins	Cephalexin (30 µg)	10 (11.1%; 5.3–20.4%)
	Cefazolin (30 µg)	5 (5.6%; 1.8–13.0%)
	Cefquinome (30 µg)	3 (3.3%; 0.7–9.7%)
	Cefotaxime (30 µg)	10 (11.1%; 5.3–20.4%)
Fluoroquinolones	Enrofloxacin (5 µg)	10 (11.1%; 5.3–20.4%)
	Marbofloxacin (5 µg)	13 (14.4%; 7.7–24.7%)
	Pradofloxacin (5 µg)	11 (12.2%; 6.1–21.9%)
Aminoglycosides	Gentamicin (10 µg)	6 (6.7%; 2.4–14.5%)
	Amikacin (30 µg)	2 (2.2%; 0.3–8.0%)
Tetracyclines	Tetracycline (30 µg)	30 (33.3%; 22.5–47.6%)
	Minocycline (30 µg)	47 (52.2%; 38.4–69.4%)
	Doxycycline (30 µg)	18 (20.0%; 11.8–31.6%)
Amphenicols	Chloramphenicol (30 µg)	10 (11.1%; 5.3–20.4%)
Macrolides	Erythromycin (15 µg)	26 (28.9%; 18.9–42.3%)
	Azithromycin (15 µg)	27 (30.0%; 19.8–43.7%)
Lincosamides	Clindamycin (2 µg)	15 (16.7%; 9.3–27.5%)
Glycopeptides	Vancomycin (30 µg)	7 (7.8%; 3.1–16.0%)
Sulphonamides	Sulfamethoxazole/trimethoprim (23.75/1.25 µg)	32 (35.6%; 24.3–50.2%)
Ansamycins	Rifampin (5 µg)	1 (1.1%; 0.03–6.1%)
Nitrofuran derivatives	Nitrofurantoin (100 µg)	32 (35.6%; 24.3–50.2%)

**Table 3:** Distribution of phenotypically methicillin-resistant commensal *Staphylococcus* sp. isolates depending on minimal inhibitory concentration of tested antimicrobial drugs.

MIC (µg/mL)	0.06	0.12	0.25	0.5	1	2	4	8	16	32	64	128	MIC_90_ (µg/mL)
Oxacillin *^,&^					3	3	9						4
Penicillin G *	2		1			2	3		6				16
Ampicillin **			2		1	2	3	2	5				16
Amoxicillin/clavulanic acid **			1	3	2	2	6		1				4
Imipenem *				11		1	2	1					4
Cephalothin **					12		1	2					8
Cefpodoxime **					1		4	2	8				16
Cefazolin **					7		2	6					8
Cefovecin **					3		3	4	5				16
Clindamycin **			3		1		1	10					8
Gentamicin *						2		7		6			32
Amikacin **								12		2	1		32
Enrofloxacin **				1	1	1	3	9					8
Marbofloxacin **				1		2		12					8
Pradofloxacin **				3	1	10	2						4
Erythromycin *		1			2	1		11					8
Tetracycline **		1		2		12							2
Doxycycline **	2		1		12								1
Minocycline **			6		4		4	1					4
Chloramphenicol *							9		1	2	3		64
Rifampin *					14		1						1
Trimethoprim/sulfamethoxazole *					2		3	10					8
Nitrofurantoin *								8		1	4	2	128
Vancomycin *				8		4	1			2			32
MIC (µg/mL)	0.06	0.12	0.25	0.5	1	2	4	8	16	32	64	128	

Note: * Human breakpoints [12]; ** veterinary breakpoints [13]; ^&^ the oxacillin resistance breakpoint for *S. aureus* is ≥4 µg/mL and ≥1 µg/mL for all other staphylococci. Concentrations corresponding to white cells for a given AMD are not included in the Sensititre plate. Colour cells correspond to sensitive (green), intermediate (yellow) and resistant (red) ranges. Isolates with MIC value above the greatest tested concentration are assigned to the next serial concentration. The MIC values of amoxicillin and trimethoprim are used for the combinations amoxicillin/clavulanic acid and trimethoprim/sulfamethoxazole, respectively.

## Data Availability

The data presented in this study are available upon request from the corresponding authors.

## Supplementary Materials

The following supporting information can be downloaded at: https://www.mdpi.com/article/10.3390/antibiotics15060536/s1, Table S1. Species diversity of staphylococci (n = 90) from healthy dogs according to isolation body site.

## Abbreviations

The following abbreviations are used in this manuscript:

AMD	antimicrobial drugs
AMR	antimicrobial resistance
CoNS	coagulase-negative staphylococci
CoPS	coagulase-positive staphylococci
MALDI-TOF	matrix-assisted laser desorption/ionization time-of-flight
MDR	multidrug-resistant
MIC	minimum inhibitory concentration
MR	methicillin-resistant
MRSA	methicillin-resistant *S. aureus*
MRSP	methicillin-resistant *S. pseudintermedius*
MSS	methicillin-sensitive staphylococci
Phen-MRSA	phenotypically methicillin-resistant *S. aureus*
Phen-MRSP	phenotypically methicillin-resistant *S. pseudintermedius*
