# Supplementary material for: Species Diversity and Antimicrobial Susceptibility of Staphylococci Colonising Healthy Dogs—A Single-Centre Cross-Sectional Study in Bulgaria
Source: Antibiotics (Basel). 2026 May 25;15(6):536. doi: 10.3390/antibiotics15060536 (PMC13296313; doi:10.3390/antibiotics15060536)
Supplement: Supplementary file 1 [file antibiotics-15-00536-s001.zip › antibiotics-4338391-supplementary.pdf]

Table S1. Species diversity of staphylococci (n=90) from healthy dogs according to isolation body site.

Species	Axilla	Groin	Ear	Anus	Vulva/ prepuce	Nose	Eye	Buccal cavity	Total
Staphylococcus pseudintermedius	7	7	5	8	2/2	8	5	5	**49**
Staphylococcus aureus	1			1	0/1	2			**5**
Staphylococcus delphini	1		1						**2**
Staphylococcus haemolyticus	3	2			0/2		1		**8**
Staphylococcus epidermidis	1	1	3					2	**7**
Staphylococcus simulans		2	1	1			1		**5**
Staphylococcus warneri		1	1			1			**3**
Staphylococcus hominis		1		1					**2**
Staphylococcus lugdunensis			1	1					**2**
Staphylococcus ureilyticus		1						1	**2**
Staphylococcus caprae	1								**1**
Staphylococcus saprophyticus	1								**1**
Staphylococcus schleiferi				1					**1**
Staphylococcus simiae		1							**1**
Staphylococcus succinus							1		**1**
**Total**	**15**	**16**	**12**	**13**	**7**	**11**	**8**	**8**	**90**
